# Annual acknowledgement of manuscript reviewers

**DOI:** 10.1186/1472-6807-14-6

**Published:** 2014-01-30

**Authors:** Simon Harold

**Affiliations:** 1 BioMed Central, Floor 6, 236 Gray’s Inn Road, London WC1X 8HB, UK

## Abstract

**Contributing reviewers:**

The editors of BMC Structural Biology would like to thank all of our reviewers who have contributed to the journal in Volume 13 (2013).

## 

Laila Abouzeid

Kuwait

Vahid Abrishami

Spain

Nelson Alves

Brazil

Jean Armengaud

France

Shannon Au

Hong Kong

Syed Sikander Azam

Pakistan

Edward Baker

New Zealand

Branimri Bertoša

Croatia

Penny Beuning

USA

Ramachandra M Bhaskara

India

José Román Bilbao-Castro

Spain

Laura Bonati

Italy

David Burke

UK

Konrad Büssow

Germany

Amedeo Caflisch

Switzerland

Guido Capitani

Switzerland

Teresa Carlomagno

Germany

Vincenzo Carnevale

USA

Hue Sun Chan

Canada

Prem P Chapagain

USA

Artem Cherkasov

Canada

Kiattawe Chuwongkomol

Thailand

Philip Cole

USA

Patricia Coltri

Brazil

Brian Dalrymple

Australia

Nicholas Dixon

Australia

Angelo Facchiano

Italy

Piero Fariselli

Italy

Deborah Fass

Israel

Natalie Fedorova

USA

Howard Feldman

Canada

Miguel Fernandes

Portugal

Federico Fogolari

Italy

Jonathan Fuller

Germany

Yonggui Gao

Singapore

Béatrice Golinelli-Pimpaneau

France

Celia Goulding

USA

Hugo Gutierrez De Teran

Sweden

Manuela Helmer-Citterich

Italy

Dominique Housset

France

Jianping Huang

China

Yin-Fu Huang

Taiwan

Thomas Huber

USA

Robert Jernigan

USA

Colin Johnson

USA

Gozde Kar

UK

Andrey Karshikoff

Bulgaria

Koichi Kato

Japan

Seiichiro Kishishita

Japan

Isabelle Krimm

France

Andriy Kryshtafovych

USA

Inari Kursula

Germany

Nicolas Leulliot

France

Shuxing Li

USA

Yeong-Shin Lin

Taiwan

Xin Liu

USA

Peer Lukat

Germany

Sethu Madhavan

USA

Giovanni Maga

Italy

Shawn Martin

New Zealand

Cristian Micheletti

Italy

Peer Mittl

Switzerland

Kenji Mizuguchi

Japan

Debasisa Mohanty

India

Hugo L Monaco

Italy

Luca Monticelli

France

Dino Moras

France

Lennart Nilsson

Sweden

Megan O'Mara

Australia

Takuji Oyama

Japan

Dev Mani Pandey

India

V Parissi

France

Stefano Pascarella

Italy

Yingjie Peng

USA

Riccardo Percudani

Italy

Stephen Perkins

UK

William Picking

USA

Stefano Pieraccini

Italy

Heather Pinkett

USA

Aina W Ravna

Norway

S. James Remington

USA

Dagmar Ringe

USA

Peter Roach

UK

Daniel Roche

France

François Rodolphe

France

Tod Romo

USA

Steven Rosenfeld

USA

Giulia Rossetti

Italy

Marc Ruff

France

Jussi Salmi

Finland

Martin Scanlon

Australia

Eric Schreiter

USA

Gary Shaw

Canada

Toshiyuki Shimizu

Japan

Blake Simmons

USA

Vasiliki Skamnaki

Greece

Joanna Slusky

USA

Carlos Oscar Sorzano

Spain

Jiri Sponer

Czech Republic

Barry Stoddard

USA

Kikue Tachibana-Konwalski

Austria

Yutaka Tamura

Japan

Yun Tang

China

Ozlem Tastan Bishop

South Africa

Colin Thorpe

USA

Guido Tiana

Italy

Sanja Tomic

Croatia

Herman Van Tilbeurgh

France

Qingguo Wang

USA

Yeming Wang

USA

Mark Wass

UK

Carrie Wilmot

USA

Dek Woolfson

UK

Ting Xu

USA

Yanhui Xu

China

Qin Yang

USA

Wei Yang

USA

Adam Yuan

Singapore

Wyatt Yue

UK

Andrea Zen

Italy

Piotr Zielenkiewicz

Poland

Wangmeng Zuo

China

